# Real‐world safety and effectiveness of guselkumab in patients with psoriasis: A post‐marketing surveillance study through up to week 52 in Japan

**DOI:** 10.1111/1346-8138.17710

**Published:** 2025-03-29

**Authors:** Yayoi Tada, Yukako Sugiura, Manami Kamishima, Shoya Takahashi, Yoshihito Tanaka, Junya Masuda, Keiichi Yamanaka

**Affiliations:** ^1^ Department of Dermatology Teikyo University Tokyo Japan; ^2^ Medical Affairs Division Johnson & Johnson Tokyo Japan; ^3^ Japan Safety & Surveillance Division Johnson & Johnson Tokyo Japan; ^4^ Statistics & Decision Sciences Division Johnson & Johnson Tokyo Japan; ^5^ Department of Dermatology Mie University Mie Japan

**Keywords:** adverse drug reaction, interleukin‐23, monoclonal antibody, post‐marketing product surveillance, treatment adherence

## Abstract

Guselkumab is a monoclonal antibody that binds to the p19 subunit of interleukin‐23 and inhibits its downstream signaling. The safety profile of guselkumab and its superior efficacy over placebo and adalimumab for the treatment of patients with moderate‐to‐severe psoriasis were reported in phase 3 studies conducted within and outside Japan. To assess the real‐world safety and effectiveness of guselkumab in Japanese patients with psoriasis, we conducted a multicenter, single‐arm, prospective, post‐marketing surveillance study. Guselkumab was administered by subcutaneous injection at a dose of 100 mg at weeks 0 and 4, then every following 8 weeks. The patient observation period was 52 weeks after the initial guselkumab dose or until treatment withdrawal. The safety analysis set consisted of 416 patients, including 310 patients with vulgaris (PsV); and the effectiveness analysis set consisted of 251 patients, including 236 patients with PsV or psoriatic arthritis (PsA). There were more men (71.3%, 221/310) than women among the PsV group. The median age among those with PsV was 58 years, the median disease duration was 11.50 years, 50.0% (155/310) had comorbidity, and 41.3% (128/310) had previously been treated with biologic agents. During the observation period, 8.4% (35/416) of patients experienced 49 adverse drug reactions, 2.9% (12/416) experienced 13 serious adverse drug reactions, and 3.4% (14/416) experienced 16 adverse events leading to treatment discontinuation. In the effectiveness analysis set of 236 patients with PsV or PsA, the Psoriasis Area and Severity Index (PASI) 75, 90, and 100 response rates at week 52 were 69.9%, 54.5%, and 32.5%, respectively. Bio‐naïve patients consistently had higher PASI 75 and 90 response rates than bio‐experienced patients. This post‐marketing surveillance study demonstrated that guselkumab was well‐tolerated and effective in a real‐world setting in Japanese patients with psoriasis.

## 1 INTRODUCTION

Psoriasis is a chronic, inflammatory skin disease and its pathogenesis is not fully understood.^1^ The most common variant is plaque‐type psoriasis or psoriasis vulgaris (PsV), which is characterized by erythematous scaly plaques often occurring on extensor surfaces.^1^ Other less common variants based on clinical presentation include guttate psoriasis, pustular psoriasis (the widespread version is called generalized pustular psoriasis [GPP]), and erythrodermic psoriasis (EP).^1^ Patients with psoriasis also develop comorbid psoriatic arthritis (PsA) involving the joints, entheses, or spine.^2^ Census data from 2010 suggest that the prevalence of psoriasis in Japan is 0.34%,^3^ lower than the figure reported for European countries.^4^

Treatments for psoriasis consist of topical agents, phototherapy (ultraviolet irradiation), and systemic therapies (oral and biologic agents).^1^ Japanese guidance recommends biologic agents for patients with moderate‐to‐severe PsV who are refractory or intolerant to other systemic therapies, patients with PsA who are at risk of irreversible joint destruction, and appropriate patients with GPP or EP.^5^ In Japan, following the approval of two anti‐tumor necrosis factor‐𝛂 inhibitors—infliximab and adalimumab—in 2010, more than 10 biologic agents are currently available for psoriasis treatment.^5^ For physicians and patients to make better treatment choices, it is essential to understand the benefits and risks of each biologic agent and the patient factors affecting drug responses.

Guselkumab is a monoclonal antibody that binds to the p19 subunit of interleukin (IL)‐23. It inhibits the downstream IL‐23 signaling pathway that promotes survival and expansion of IL‐17‐producing T helper cells, which produce pro‐inflammatory effector molecules.^6^ The safety profile of guselkumab and its superior efficacy compared with placebo or adalimumab for the treatment of patients with moderate‐to‐severe psoriasis were reported in two global, double‐blind, phase 3 studies (VOYAGE 1^7^ and VOYAGE 2^8^). In two phase 3 studies conducted in Japan, guselkumab showed efficacy and safety in patients with moderate‐to‐severe PsV^9^ as well as those with PsA, GPP^10^ or EP. On the basis of these results, guselkumab was approved in Japan in 2018 for treatment of psoriasis that is refractory to other approved treatment options.^11^ Most previous clinical studies excluded patients who had recently been treated with traditional systemic therapies, phototherapy, or biologic agents,^7^, ^8^, ^9^, ^10^ and it is, therefore, essential to assess the real‐world safety and effectiveness profile of guselkumab in patients who, for various reasons, need to switch from their previous treatment.

We, therefore, conducted a prospective, post‐marketing, surveillance study of guselkumab in Japanese patients with PsV, PsA, GPP, or EP. The interim results at week 20 of treatment were reported previously.^12^ This paper reports the real‐world safety and effectiveness of guselkumab up to 52 weeks, focusing on the impact of patient factors on responses to treatment.

## 2 METHODS

### 2.1 Study design

We conducted a multicenter, single‐arm, prospective post‐marketing, surveillance study to assess the real‐world safety and effectiveness of guselkumab in Japanese patients with psoriasis. The observation period for each patient was 52 weeks after the initial guselkumab injection or until treatment withdrawal. Guselkumab was administered by subcutaneous injection at a dose of 100 mg at weeks 0 and 4, then every following 8 weeks. The protocol and ethical considerations of the study were approved by the internal review board of each participating institution. The study was conducted in accordance with Good Post‐marketing Study Practice ([GPSP] the Ministry of Health, Labour and Welfare Ordinance No. 171., dated December 20, 2004), which does not require patient consent for post‐marketing, surveillance studies.

### 2.2 Patients

Japanese patients with PsV, PsA, GPP, or EP were registered within 14 days of their initial guselkumab injection if they had started guselkumab for the first time between May 2018 and October 2020 at participating institutions.

### 2.3 Data collection and endpoints

Patient baseline characteristics, including psoriasis type, psoriasis severity, medical history, and comorbidity, were collected at registration. Information on guselkumab treatment and concomitant drugs was collected throughout the observation period.

We collected information on adverse events (AEs), serious AEs, adverse drug reactions (ADRs), and serious ADRs throughout the observation period for safety assessments. ADRs are AEs for which a causal relationship to drug treatment could not be ruled out by the investigator or the study sponsor. AEs were coded by System Organ Class and Preferred Term using the Medical Dictionary for Regulatory Activities, version 26.0. Serious infections, serious hypersensitivity reactions, malignancy, decreased neutrophil count, and major adverse cardiovascular events were considered safety topics of interest.

We measured the following parameters for the effectiveness assessment: Psoriasis Area and Severity Index (PASI; ranging from 0 to 72; a higher score indicates more severe psoriasis),^13^ clinical global impression (CGI) evaluated by the treating physicians (very much improved, much improved, minimally improved, no change, and worse),^10^, ^14^ Dermatology Life Quality Index (DLQI; ranging from 0 to 30; a higher score shows greater impairment of quality of life),^15^ Disease Activity Score 28 (DAS28) using C‐reactive protein (CRP),^16^ and patient global assessment of disease activity (PtGA).^17^ PASI 75, 90, and 100 response rates were defined as the percentage of patients with ≥75%, ≥90%, and 100% reduction respectively in their PASI score from baseline. Absolute PASI score was also used to assess achievement of treatment goals.^18^

### 2.4 Statistical analysis

The safety analysis set consisted of patients eligible for the study, excluding those with protocol violations or incomplete initial case report forms. The effectiveness analysis set consisted of patients in the safety analysis set, except for those with missing or incomplete data on effectiveness assessments. Changes in PASI and DLQI scores from baseline were assessed using the paired *t*‐test without multiplicity adjustment. The association of each patient factor with the occurrence of ADRs or the achievement of a PASI 90 response was assessed by estimating the odds ratios (ORs) and 95% confidence intervals (CIs) using univariable and multivariable logistic regression analyses. The Wald test was used to calculate *p‐*values for ORs. Correlation analyses were performed between the change in PASI score from baseline and disease duration. A patient's treatment was considered to have been successful if their CGI ratings were “very much improved,” “much improved,” or “minimally improved.”^10^ Missing data were not imputed. Unless otherwise specified, a *p*‐value <0.05 was considered statistically significant in two‐tailed tests without multiplicity adjustment in this study. All statistical analyses used SAS version 9.4 (SAS Institute Inc.).

## 3 RESULTS

### 3.1 Patients

**FIGURE 1:**
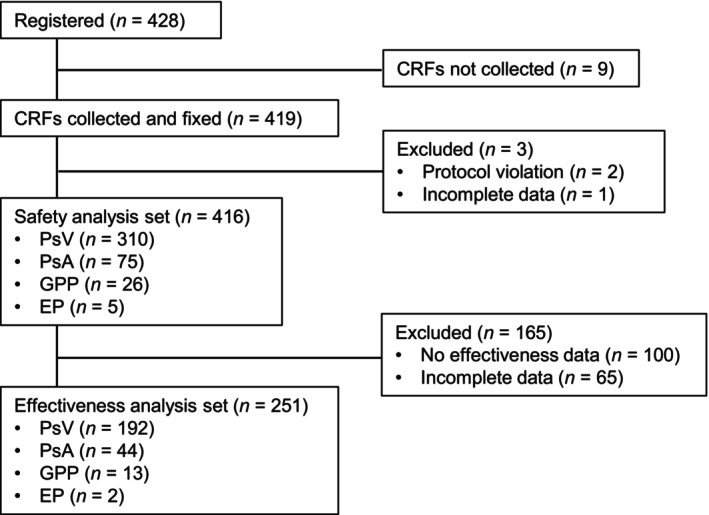
Patient flowchart. Of the 428 patients with psoriasis registered in the study, 416 were included in the safety analysis set, and 251 were included in the effectiveness analysis set. CRF, case report form; EP, erythrodermic psoriasis; GPP, generalized pustular psoriasis; PsA, psoriatic arthritis; PsV, psoriasis vulgaris.

A total of 428 patients with psoriasis were registered and case report forms were collected from 419 patients (Figure 1). The safety analysis set consisted of 416 patients after excluding three patients (protocol violation, *n* = 2; incomplete data, *n* = 1), and the effectiveness analysis set consisted of 251 patients after excluding 165 patients (no effectiveness data, *n* = 100; incomplete data, *n* = 65).

Patients with PsV made up 74.5% (310/416) of those in the safety analysis set (Table 1). There were more men than women in the PsV (71.3%, 221/310), the PsA (52.0%, 39/75) and EP (100.0%, 5/5) groups, but more women in the GPP group (65.4%, 17/26). The median age was 58 years in the PsV and PsA groups, 60 years in the GPP group, and 63 years in the EP group. The median disease duration was 11.50 years in the PsV group, 13.00 years in the PsA group, 7.25 years in the GPP group, and 14.00 years in the EP group. The median PASI and DLQI scores were 10.5 and 6 in the PsV group, 7.4 and 6 in the PsA group, 6.2 and 3 in the GPP group, and 21.5 and 12 in the EP group. The percentages of patients who had a history of a prior medical condition or comorbidity were 20.6% (64/310) and 50.0% (155/310) in the PsV group, 22.7% (17/75) and 65.3% (49/75) in the PsA group, 34.6% (9/26) and 69.2% (18/26) in the GPP group, and 40.0% (2/5) and 40.0% (2/5) in the EP group. The percentages of patients who were previously treated with one or more biologic agent and who had concomitant treatment were 41.3% (128/310) and 66.5% (206/310) in the PsV group, 62.7% (47/75) and 76.0% (57/75) in the PsA group, 73.1% (19/26) and 65.4% (17/26) in the GPP group, and 20.0% (1/5) and 100.0% (5/5) in the EP group.

**TABLE 1:** Baseline characteristics of patients (safety analysis set, *n* = 416).

	PsV *n* = 310	PsA *n* = 75	GPP *n* = 26	EP *n* = 5
Sex, male	221 (71.3)	39 (52.0)	9 (34.6)	5 (100.0)
Age, years				
Median (min–max)	58 (18–97)	58 (36–81)	60 (26–88)	63 (18–74)
Mean (SD)	57.8 (15.6)	58.2 (11.9)	58.8 (17.0)	55.8 (22.0)
<65	191 (61.6)	53 (70.7)	14 (53.8)	3 (60.0)
≥65	119 (38.4)	22 (29.3)	12 (46.2)	2 (40.0)
Weight, kg, median (min–max)^a^	67.0 (40.0–130.0)	65.0 (38.0–139.5)	58.0 (32.0–87.0)	64.0 (57.0–70.9)
Disease duration, years				
Median (min–max)^b^	11.50 (0.08–50.00)	13.00 (0.50–50.00)	7.25 (0.08–40.00)	14.00 (0.42–36.00)
Mean (SD)^b^	13.81 (9.78)	15.07 (12.44)	12.26 (12.43)	14.48 (14.56)
<10	103 (33.2)	31 (41.3)	16 (61.5)	2 (40.0)
≥10 and <20	106 (34.2)	21 (28.0)	3 (11.5)	1 (20.0)
≥20 and <30	57 (18.4)	13 (17.3)	4 (15.4)	1 (20.0)
≥30	28 (9.0)	9 (12.0)	3 (11.5)	1 (20.0)
Unknown	16 (5.2)	1 (1.3)	0 (0.0)	0 (0.0)
PASI				
Median (min, max)^c^	10.5 (0.0–48.5)	7.4 (0.0–35.0)	6.2 (0.0–27.0)	21.5 (20.4–49.2)
Mean (SD)^c^	12.11 (9.07)	9.57 (9.21)	8.40 (7.39)	30.37 (16.32)
≤5	50 (16.1)	20 (26.7)	5 (19.2)	0 (0.0)
>5 and ≤10	48 (15.5)	14 (18.7)	5 (19.2)	0 (0.0)
>10 and ≤20	77 (24.8)	10 (13.3)	2 (7.7)	0 (0.0)
>20	34 (11.0)	6 (8.0)	1 (3.8)	3 (60.0)
Unknown	101 (32.6)	25 (33.3)	13 (50.0)	2 (40.0)
DLQI, median (min–max)^d^	6 (0–28)	6 (0–21)	3 (0–8)	12 (2–24)
Previous medical condition, yes	64 (20.6)	17 (22.7)	9 (34.6)	2 (40.0)
Comorbidity, yes	155 (50.0)	49 (65.3)	18 (69.2)	2 (40.0)
Prior biological treatment, yes	128 (41.3)	47 (62.7)	19 (73.1)	1 (20.0)
Concomitant treatment, yes	206 (66.5)	57 (76.0)	17 (65.4)	5 (100.0)

*Note*: Values are given as *n* (%) unless otherwise noted.

Abbreviations: DLQI, dermatology life quality index; EP, erythrodermic psoriasis; GPP, generalized pustular psoriasis; PASI, psoriasis area and severity index; PsA, psoriatic arthritis; PsV, psoriasis vulgaris; SD, standard deviation.

^a^ *n* = 278 for PsV, *n* = 72 for PsA, and *n* = 23 for GPP.

^b^ *n* = 294 for PsV and *n* = 74 for PsA.

^c^ *n* = 209 for PsV, *n* = 50 for PsA, *n* = 13 for GPP, and *n* = 3 for EP.

^d^ *n* = 107 for PsV, *n* = 27 for PsA, *n* = 3 for GPP, and *n* = 3 for EP.

### 3.2 Safety

During the observation period, 8.4% (35/416) of patients in the safety analysis set experienced 49 ADRs (including 13 serious ADRs), and 3.4% (14/416) experienced 16 AEs leading to treatment discontinuation (recovered, *n* = 4; recovering, *n* = 6; unrecovered, *n* = 4; and death, *n* = 2 [lung neoplasm, malignant, *n* = 1, a causal relationship to drug treatment was ruled out; pancreatic carcinoma, *n* = 1, a causal relationship to drug treatment was not ruled out]; Table 2). Each ADR by Preferred Term was reported in <1.0% of patients in the safety analysis set (Table 3). Among safety topics of interest, serious infections were reported in four patients (aspergilloma, *n* = 1; herpes zoster, *n* = 1; pneumonia, *n* = 1; and enteritis infection, *n* = 1) and malignancies were reported in two patients (pancreatic carcinoma, *n* = 1; hepatocellular carcinoma, *n* = 1). No AEs of serious hypersensitivity reaction, decreased neutrophil count, nor major adverse cardiovascular events were reported. No patient developed tuberculosis or hepatitis B virus reactivation.

**TABLE 2:** Summary of ADRs and AEs (safety analysis set, *n* = 416).

	Total	Serious	Non‐serious
ADRs			
Number of patients	35 (8.4)	12 (2.9)	25 (6.0)
Number of events	49	13	36
AEs leading to treatment discontinuation			
Number of patients	14 (3.4)	10 (2.4)	4 (1.0)
Number of events	16^a^	12	4

*Note*: Values are given as *n* or *n* (%).

Abbreviations: ADRs, adverse drug reactions; AEs, adverse events.

^a^ Recovered (*n* = 4), recovering (*n* = 6), unrecovered (*n* = 4), and death (*n* = 2).

**TABLE 3:** Details of ADRs^a^ (safety analysis set, *n* = 416).

	Total	Serious	Non‐serious
Infections and infestations			
Aspergilloma	1 (0.2)	1 (0.2)	0 (0.0)
Furuncle	1 (0.2)	0 (0.0)	1 (0.2)
Herpes zoster	2 (0.5)	1 (0.2)	1 (0.2)
Nasopharyngitis	3 (0.7)	0 (0.0)	3 (0.7)
Oral candidiasis	1 (0.2)	0 (0.0)	1 (0.2)
Pneumonia	2 (0.5)	1 (0.2)	1 (0.2)
Tinea pedis	1 (0.2)	0 (0.0)	1 (0.2)
Tonsillitis	1 (0.2)	0 (0.0)	1 (0.2)
Enteritis infectious	1 (0.2)	1 (0.2)	0 (0.0)
Bacterial infection	1 (0.2)	0 (0.0)	1 (0.2)
Nail infection	1 (0.2)	0 (0.0)	1 (0.2)
Oral herpes	1 (0.2)	0 (0.0)	1 (0.2)
Neoplasms benign, malignant and unspecified (incl cysts and polyps)			
Pancreatic carcinoma	1 (0.2)	1 (0.2)	0 (0.0)
Hepatocellular carcinoma	1 (0.2)	1 (0.2)	0 (0.0)
Endocrine disorders			
Hyperthyroidism	1 (0.2)	1 (0.2)	0 (0.0)
Metabolism and nutrition disorders			
Hyponatremia	1 (0.2)	1 (0.2)	0 (0.0)
Vascular disorders			
Vasodilatation	1 (0.2)	0 (0.0)	1 (0.2)
Respiratory, thoracic and mediastinal disorders			
Cough	1 (0.2)	0 (0.0)	1 (0.2)
Emphysema	1 (0.2)	1 (0.2)	0 (0.0)
Interstitial lung disease	2 (0.5)	2 (0.5)	0 (0.0)
Cough variant asthma	1 (0.2)	0 (0.0)	1 (0.2)
Hepatobiliary disorders			
Hepatic function abnormal	1 (0.2)	1 (0.2)	0 (0.0)
Hyperplastic cholecystopathy	1 (0.2)	0 (0.0)	1 (0.2)
Skin and subcutaneous tissue disorders			
Acne	1 (0.2)	0 (0.0)	1 (0.2)
Alopecia	1 (0.2)	0 (0.0)	1 (0.2)
Asteatotic eczema	1 (0.2)	0 (0.0)	1 (0.2)
Erythrodermic psoriasis	1 (0.2)	1 (0.2)	0 (0.0)
Pruritus	2 (0.5)	0 (0.0)	2 (0.5)
Pyoderma gangrenosum	1 (0.2)	0 (0.0)	1 (0.2)
Urticaria	3 (0.7)	0 (0.0)	3 (0.7)
Mechanical urticaria	1 (0.2)	0 (0.0)	1 (0.2)
Musculoskeletal and connective tissue disorders			
Arthralgia	2 (0.5)	0 (0.0)	2 (0.5)
General disorders and administration site conditions			
Administration site reaction	2 (0.5)	0 (0.0)	2 (0.5)
Pyrexia	1 (0.2)	0 (0.0)	1 (0.2)
Investigations			
Blood creatine phosphokinase increased	1 (0.2)	0 (0.0)	1 (0.2)
Blood beta‐D‐glucan increased	1 (0.2)	0 (0.0)	1 (0.2)
Hepatic enzyme increased	2 (0.5)	0 (0.0)	2 (0.5)
KL‐6 increased	1 (0.2)	0 (0.0)	1 (0.2)

*Note*: Values are given as *n* (%).

Abbreviations: ADRs, adverse drug reactions, KL‐6, Krebs von den Lungen‐6.

^a^ ADRs were coded using system organ class codes and preferred term codes in the Medical Dictionary for Regulatory Activities, version 26.0.

**FIGURE 2:**
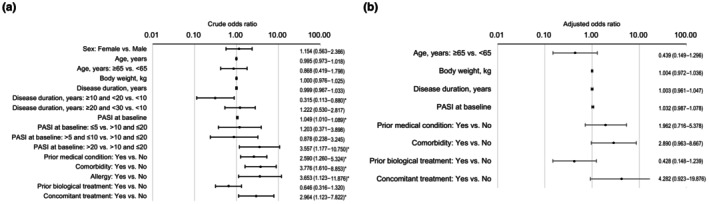
Factors associated with adverse drug reactions (safety analysis set, *n* = 416). (a) Univariable logistic regression analyses demonstrated a significant association between adverse drug reactions and disease duration categories (i.e., ≥10 years and <20 years vs <10 years), baseline Psoriasis Area and Severity Index (PASI) score, baseline PASI score categories (i.e., >20 vs >10 and ≤20), a history of a prior medical condition, presence of comorbidity, allergy, and concomitant treatment. (b) A multivariable logistic regression analysis identified no patient factors that were significantly associated with adverse drug reactions . The numbers on the right indicate the odds ratios and their 95% confidence intervals. Asterisks indicate a statistically significant association (*p* < 0.05).

Univariable logistic regression analyses showed that the risk of ADRs was lower for patients with a disease duration of ≥10 and <20 years than those with a disease duration of <10 years (OR 0.315; 95% CI 0.113–0.880; *p* = 0.027), and higher for patients with a higher baseline PASI score (OR per unit increase 1.049; 95% CI 1.010–1.089; *p* = 0.012), those with a baseline PASI score >20 compared with those with a baseline PASI score >10 and ≤20 (OR 3.557; 95% CI 1.177–10.750; *p* = 0.025), and those with a history of a prior medical condition (OR 2.590; 95% CI 1.260–5.324; *p* = 0.010), comorbidity (OR 3.776; 95% CI 1.610–8.853; *p* = 0.002), allergy (OR 3.653; 95% CI 1.123–11.876; *p* = 0.031), or concomitant treatment (OR 2.964; 95% CI 1.123–7.822; *p* = 0.028) (Figure 2a). Multivariable logistic regression analysis identified no patient factor that significantly influenced the risk of ADRs (Figure 2b).

### 3.3 Effectiveness

**FIGURE 3:**
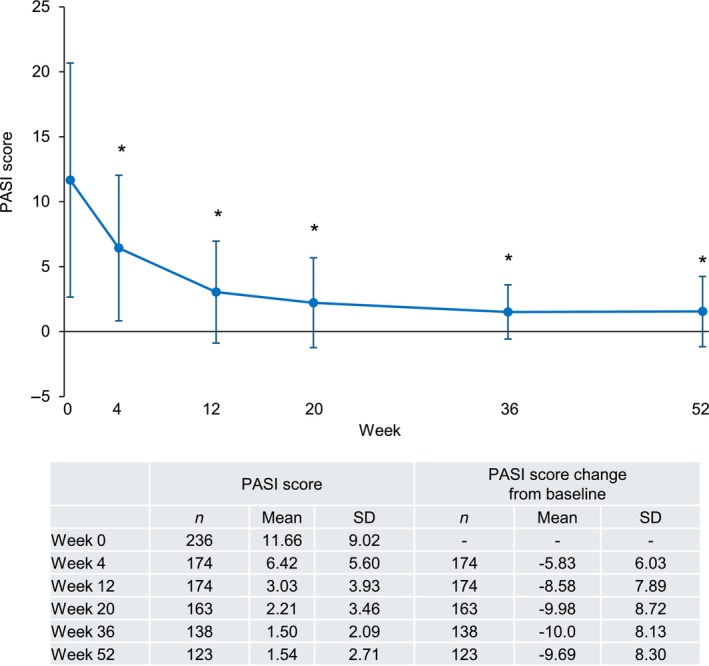
Psoriasis Area and Severity Index (PASI) scores in patients with psoriasis vulgaris (PsV) or psoriatic arthritis (PsA) (effectiveness analysis set, *n* = 236). The mean PASI score decreased after the first guselkumab injection and stayed significantly lower than the baseline value through to week 52. Error bars indicate standard deviation. Asterisks indicate a statistically significant decrease from baseline (*p* < 0.05). SD, standard deviation.

**FIGURE 4:**
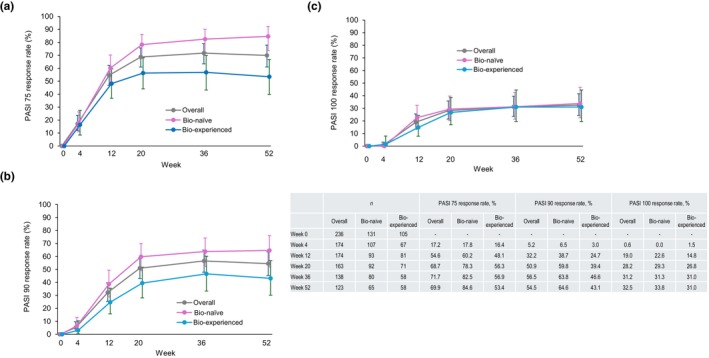
Psoriasis Area and Severity Index (PASI) response rates in patients with psoriasis vulgaris (PsV) or psoriatic arthritis (PsA) stratified by prior biologic agent use (effectiveness analysis set, *n* = 236). (a–c) PASI 75, 90, and 100 response rates increased after the first guselkumab injection and reached a plateau at week 20. (a, b) Bio‐naïve patients consistently had higher PASI 75 and 90 response rates than bio‐experienced patients. Error bars indicate 95% confidence interval.

In patients with PsV or PsA, the PASI score decreased after the first guselkumab injection and stayed significantly lower than the baseline value (between −5.83 and −10.0 from baseline in mean) through to week 52 (Figure 3). Similarly, PASI 75, 90, and 100 response rates increased after the first guselkumab injection and reached a plateau at week 20 (Figure 4a–c). PASI 75, 90, and 100 response rates (95% CI) in the overall population at week 52 were 69.9% (61.0%–77.9%), 54.5% (45.2%–63.5%), and 32.5% (24.4%–41.6%), respectively. Bio‐naïve patients consistently had higher PASI 75 and 90 response rates than bio‐experienced patients (Figure 4a,b). At week 52, PASI 75 response rates (95% CI) were 84.6% (73.5%–92.4%) in bio‐naïve and 53.4% (39.9%–66.7%) in bio‐experienced patients (Figure 4a) and PASI 90 response rates were 64.6% (51.8%–76.1%) in bio‐naïve and 43.1% (30.2%–56.8%) in bio‐experienced patients (Figure 4b). Comparable PASI 100 response rates were observed and were 33.8% (95% CI 22.6%–46.6%) in bio‐naïve and 31.0% (95% CI 19.5%–44.5%) in bio‐experienced patients (Figure 4c).

**FIGURE 5:**
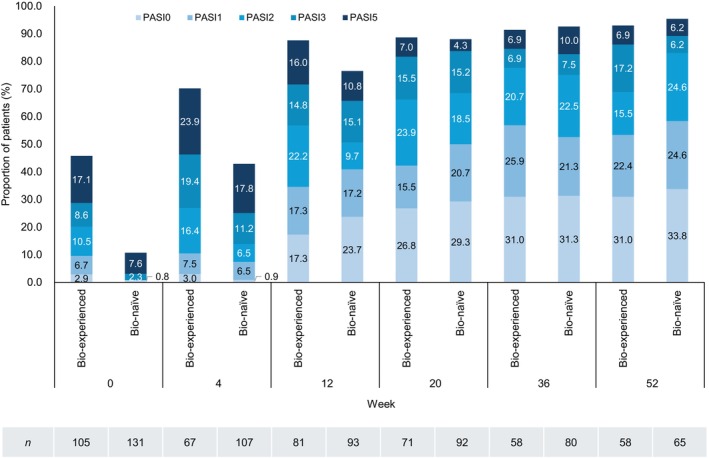
Achievement of absolute Psoriasis Area and Severity Index (PASI) responses in patients with psoriasis vulgaris (PsV) or psoriatic arthritis (PsA) stratified by prior biologic agent use (effectiveness analysis set, *n* = 236). Bio‐naïve patients had higher baseline absolute PASI scores than bio‐experienced patients; however, comparable absolute PASI responses were achieved over time.

Among patients with PsV or PsA, the percentages of bio‐experienced and bio‐naïve patients with an absolute PASI score ≤5 were 45.8% and 10.7% at week 0, 87.6% and 76.5% at week 12, and 93.0% and 95.4% at week 52. These findings indicate that bio‐naïve patients had a more severe disease than bio‐experienced patients at baseline but achieved a similar level of disease control over time (Figure 5). Similarly, the percentages of bio‐experienced and bio‐naïve patients with an absolute PASI score ≤1 were 9.5% and 0.8% at week 0, 34.6% and 40.9% at week 12, and 53.4% and 58.5% at week 52 (Figure 5).

**FIGURE 6:**
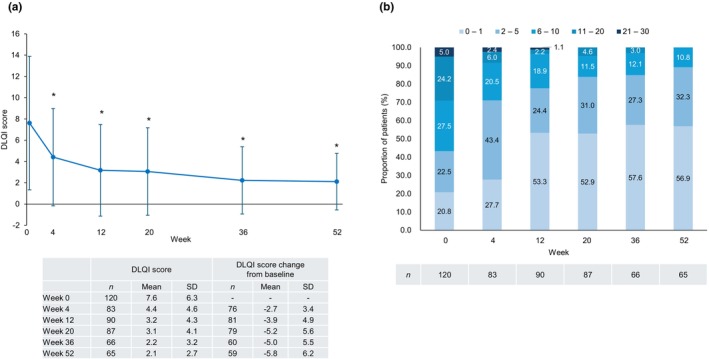
Quality of life in patients with psoriasis vulgaris (PsV) or psoriatic arthritis (PsA) (effectiveness analysis set, *n* = 120). (a) The mean Dermatology Life Quality Index (DLQI) score decreased after the first guselkumab injection and remained significantly lower than the baseline value through to week 52. (b) Nearly 90% of patients achieved a DLQI score of ≤5 at week 52. Error bars indicate standard deviation (SD). Asterisks indicate a statistically significant decrease from baseline (*p* < 0.05).

In patients with PsV or PsA, the DLQI score decreased after the first guselkumab injection and remained significantly lower than the baseline value through to week 52 (Figure 6a). Nearly 90% of patients achieved a DLQI score ≤5 at week 52 (Figure 6b).

**FIGURE 7:**
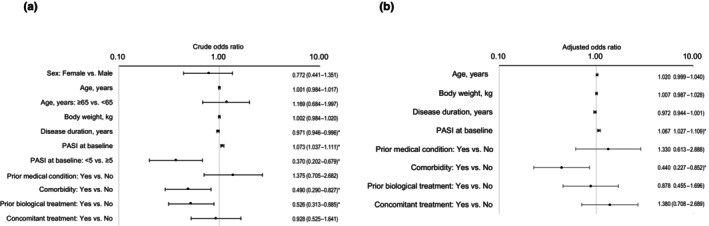
Factors associated with achieving a Psoriasis Area and Severity Index (PASI) 90 response in patients with psoriasis vulgaris (PsV) or psoriatic arthritis PsA (effectiveness analysis set, *n* = 236). (a) Univariable logistic regression analyses demonstrated a significant association between achieving a PASI 90 response and disease duration, baseline PASI score, baseline PASI score categories (i.e., < 5 vs ≥ 5), presence of comorbidity, and prior biological treatment. (b) A multivariable logistic regression analysis demonstrated a significant association between achieving a PASI 90 response and baseline PASI score and presence of comorbidity. The numbers on the right indicate the odds ratios and their 95% confidence intervals. Asterisks indicate a statistically significant association (*p* < 0.05).

Univariable logistic regression analyses determined that the probability of achieving a PASI 90 response was higher for patients with a higher baseline PASI score (OR per unit increase 1.073; 95% CI 1.037–1.111; *p* < 0.001), and lower for patients with longer disease duration (OR per year increase 0.971; 95% CI 0.946–0.996; *p* = 0.022), a baseline PASI score <5 compared with ≥5 (OR 0.370; 95% CI 0.202–0.679; *p* = 0.001), and comorbidity (OR 0.490; 95% CI 0.290–0.827; *p* = 0.008) or prior biological treatment (OR 0.526; 95% CI 0.313–0.885; *p* = 0.015) (Figure 7a). Multivariable logistic regression analysis showed that the probability of achieving a PASI 90 response was greater in patients with a higher baseline PASI score (OR per unit increase 1.067; 95% CI 1.027–1.109; *p* = 0.001) and lower in patients with any comorbidity compared with none (OR 0.440; 95% CI 0.227–0.852; *p* = 0.015) (Figure 7b).

**FIGURE 8:**
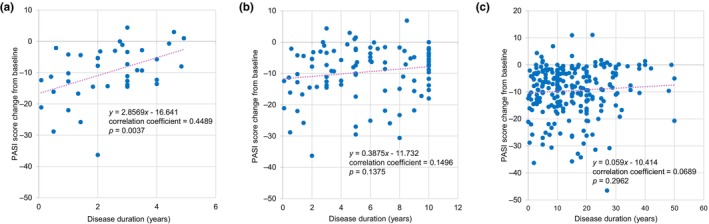
Correlation between disease duration and change from baseline in Psoriasis Area and Severity Index (PASI) score at week 52 in patients with psoriasis vulgaris (PsV) or psoriatic arthritis PsA (effectiveness analysis set). (a) Correlation analyses demonstrated a significant positive correlation between change from baseline in PASI score at week 52 and disease duration in patients with a disease duration of <5 years (b, c) but not in those with disease duration of ≤10 years or in the overall patient population. Dotted lines indicate regression lines.

In patients with PsV or PsA, the overall prevalence of any comorbidity and cardiometabolic comorbidity (hypertension, diabetes mellitus, hyperuricemia, dyslipidemia, or hyperlipidemia) was 55.9% (132/236) and 32.2% (76/236), all of which increased with disease duration (Table S1). A positive correlation between change from baseline in PASI score at week 52 and disease duration was seen among patients with a disease duration of <5 years (correlation coefficient 0.4489, *p* = 0.0037) (Figure 8a), but not those with a disease duration of ≤10 years or any disease duration (Figure 8b,c).

**FIGURE 9:**
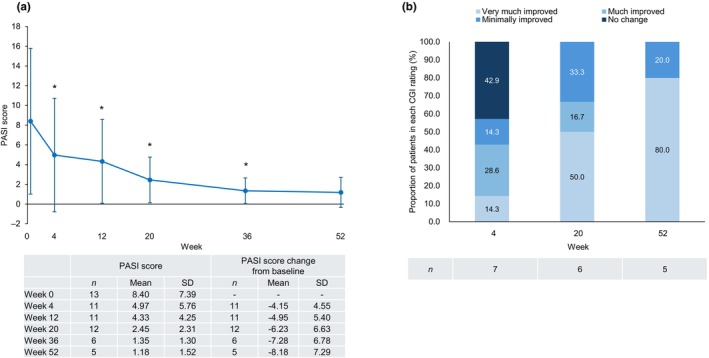
Effectiveness of guselkumab in patients with generalized pustular psoriasis (GPP) (effectiveness analysis set, *n* = 13). (a) Mean Psoriasis Area and Severity Index (PASI) score decreased after the first guselkumab injection and remained significantly lower than the baseline value through week 36. (b) The percentage of patients with a clinical global impression (CGI) rating of “very much improved” or “minimally improved” was 100.0% at week 52. Error bars indicate standard deviation (SD). Asterisks indicate a statistically significant decrease from baseline (*p* < 0.05).

In patients with GPP, the PASI score decreased after the first guselkumab injection and remained significantly lower than baseline through to week 36 (Figure 9a). The percentage of patients treated successfully (treatment success), based on achieving a CGI rating of “very much improved,” “much improved,” or “minimally improved,” reached 100.0% at week 52 (Figure 9b).

In patients with PsA, the DAS28 (CRP) score was significantly lower than baseline at week 20 (mean change from baseline of −0.69) and numerically lower than baseline at week 52 (mean change from baseline of −0.71) (Table S2‐1). Based on the mean DAS28 (CRP) score,^19^ patients' disease activity improved from moderate (3.10) at baseline to low (2.34) at week 52. The PtGA score was also significantly lower than baseline at week 20 (mean change from baseline of −25.00), and numerically lower than baseline at week 52 (mean change from baseline of −22.57) (Table S2‐2).

### 3.4 Treatment persistence

The treatment discontinuation rate at week 52 was 26.9% (112/416) in the overall population, 24.8% (77/310) in the PsV group, 34.7% (26/75) in the PsA group, 30.8% (8/26) in the GPP group, and 20.0% (1/5) in the EP group (Table 4). The timing of discontinuation varied throughout the observation period, but most often occurred after week 4 in the PsV group, between weeks 4 and 36 in the PsA group, between weeks 20 and 36 in the GPP group, and before week 4 in the EP group.

**TABLE 4:** Treatment discontinuation rate, stratified by psoriasis type (safety analysis set).

	*n* ^a^	Total discontinuation	Weeks				
			<4	≥4 and <12	≥12 and <20	≥20 and <36	≥36
Overall	416	112 (26.9)	9 (2.2)	28 (6.7)	20 (4.8)	34 (8.2)	21 (5.0)
PsV	310	77 (24.8)	4 (1.3)	21 (6.8)	12 (3.9)	22 (7.1)	18 (5.8)
PsA	75	26 (34.7)	4 (5.3)	6 (8.0)	7 (9.3)	7 (9.3)	2 (2.7)
GPP	26	8 (30.8)	0 (0.0)	1 (3.8)	1 (3.8)	5 (19.2)	1 (3.8)
EP	5	1 (20.0)	1 (20.0)	0 (0.0)	0 (0.0)	0 (0.0)	0 (0.0)

*Note*: Values are given as *n* or *n* (%).

Abbreviations: EP, erythrodermic psoriasis; GPP, generalized pustular psoriasis; PsA, psoriatic arthritis; PsV, psoriasis vulgaris.

^a^ Patients with outcome data by week 52.

In patients with PsV or PsA, the treatment discontinuation rate at week 52 was 28.6% (50/175) in patients who had previously been treated with biologic agents and 25.2% (53/210) in those who had not (Table 5). The most common reasons for treatment discontinuation in patients with PsV or PsA were insufficient effect (*n* = 25), patient's request (*n* = 15), and AE (*n* = 4) in bio‐experienced patients, and no‐show (*n* = 17), insufficient effect (*n* = 13), and patient's request (*n* = 10) in bio‐naïve patients (Table 6).

**TABLE 5:** Treatment discontinuation rate in patients with PsV or PsA, stratified by prior biologic treatment (safety analysis set).

	*n* ^a^	Total discontinuation	Weeks				
			<4	≥4 and <12	≥12 and <20	≥20 and <36	≥36
PsV or PsA	385	103 (26.8)	8 (2.1)	27 (7.0)	19 (4.9)	29 (7.5)	20 (5.2)
Bio‐experienced	175	50 (28.6)	3 (1.7)	13 (7.4)	9 (5.1)	17 (9.7)	8 (4.6)
Bio‐naïve	210	53 (25.2)	5 (2.4)	14 (6.7)	10 (4.8)	12 (5.7)	12 (5.7)

*Note*: Values are given as *n* or *n* (%).

Abbreviations: PsA, psoriatic arthritis; PsV, psoriasis vulgaris.

^a^ Patients with outcome data by week 52.

**TABLE 6:** Reasons for treatment discontinuation in patients with PsV or PsA (safety analysis set).

	Total discontinuation	Weeks				
		<4	≥4 and <12	≥12 and <20	≥20 and <36	≥36
Symptom improvement	4	0	0	3	0	1
Bio‐experienced	1	0	0	0	0	1
Bio‐naïve	3	0	0	3	0	0
Patient's request	25	2	11	3	5	4
Bio‐experienced	15	1	7	2	2	3
Bio‐naïve	10	1	4	1	3	1
Adverse event	9	1	2	2	2	2
Bio‐experienced	4	0	1	1	2	0
Bio‐naïve	5	1	1	1	0	2
Insufficient effect	38	3	6	9	12	8
Bio‐experienced	25	2	4	6	10	3
Bio‐naïve	13	1	2	3	2	5
Transfer to another hospital	2	0	1	0	1	0
Bio‐experienced	2	0	1	0	1	0
Bio‐naïve	0	0	0	0	0	0
No‐show	20	2	5	2	7	4
Bio‐experienced	3	0	0	0	2	1
Bio‐naïve	17	2	5	2	5	3
Other	5	0	2	0	2	1
Bio‐experienced	0	0	0	0	0	0
Bio‐naïve	5	0	2	0	2	1

*Note*: Values are given as *n*.

Abbreviations: PsA, psoriatic arthritis; PsV, psoriasis vulgaris.

In patients with PsV or PsA, the mean (standard deviation) PASI score at treatment discontinuation for any reason was 5.68 (5.69) in bio‐experienced patients and 3.18 (4.10) in bio‐naïve patients (Table 7). The mean PASI score at treatment discontinuation tended to be lower, and the negative percent change from baseline larger, for patients who discontinued treatment at their request compared with those whose treatment discontinuation was related to insufficient effect (mean PASI score 1.43 vs 6.68; mean percent change from baseline of −72.48% vs −16.25%). Among bio‐naïve patients, the mean PASI score at treatment discontinuation tended to be lower, and the negative percent change from baseline larger, in patients who discontinued treatment because of symptom improvement, patient request, or no‐show compared with insufficient effect (mean PASI score of 1.40 [symptom improvement], 0.92 [patient's request], 3.18 [no‐show], and 5.92 [insufficient effect]; mean percent change from baseline of −75.00% [symptom improvement], −91.71% [patient's request], −77.10% [no‐show], and −42.97% [insufficient effect]).

**TABLE 7:** PASI scores at treatment discontinuation in patients with PsV or PsA, stratified by reason for discontinuation (effectiveness analysis set).

	*n*	PASI score at treatment discontinuation		
		Observed value	Change from baseline	Percent change from baseline, %
Any reason	55	4.22 (4.94)	−8.02 (11.45)	−45.18 (65.46)^a^
Bio‐experienced	23	5.68 (5.69)	−4.70 (13.41)	−7.15 (77.73)^b^
Bio‐naïve	32	3.18 (4.10)	−10.41 (9.31)	−70.95 (39.47)^c^
Symptom improvement	2	1.40 (1.98)	−4.55 (2.47)	−75.00 (35.36)
Bio‐experienced	0	—	—	—
Bio‐naïve	2	1.40 (1.98)	−4.55 (2.47)	−75.00 (35.36)
Patient's request	11	1.43 (1.67)	−12.92 (10.99)	−72.48 (45.15)
Bio‐experienced	5	2.04 (1.97)	−6.54 (7.53)	−49.39 (60.68)
Bio‐naïve	6	0.92 (1.33)	−18.23 (11.03)	−91.71 (12.45)
Adverse event	5	4.64 (5.44)	−4.42 (6.97)	−41.92 (63.96)
Bio‐experienced	2	9.55 (5.16)	2.10 (1.70)	25.67 (10.84)
Bio‐naïve	3	1.37 (2.37)	−8.77 (4.99)	−86.98 (22.54)
Insufficient effect	24	6.68 (5.64)	−4.27 (11.63)	−16.25 (73.62)^d^
Bio‐experienced	14	7.21 (6.08)	−3.26 (14.39)	3.80 (81.67)^e^
Bio‐naïve	10	5.92 (5.17)	−5.69 (6.54)	−42.97 (54.54)^f^
Transfer to another hospital	2	0.20 (0.28)	−17.00 (24.32)	0.00 (141.42)
Bio‐experienced	2	0.20 (0.28)	−17.00 (24.32)	0.00 (141.42)
Bio‐naïve	0	—	—	—
No‐show	9	3.18 (3.90)	−13.28 (10.14)	−77.10 (31.84)
Bio‐experienced	0	—	—	—
Bio‐naïve	9	3.18 (3.90)	−13.28 (10.14)	−77.10 (31.84)
Other	2	0.70 (0.99)	−5.95 (5.73)	−78.79 (30.00)
Bio‐experienced	0	—	—	—
Bio‐naïve	2	0.70 (0.99)	−5.95 (5.73)	−78.79 (30.00)

*Note*: Values are given as the mean (standard deviation).

Abbreviations: PASI, Psoriasis Area and Severity Index; PsA, psoriatic arthritis; PsV, psoriasis vulgaris.

^a^ *n* = 52.

^b^ *n* = 21.

^c^ *n* = 31.

^d^ *n* = 21.

^e^ *n* = 12.

^f^ *n* = 9.

### 3.5 Treatment interruption

A total of 33 episodes of treatment interruption were reported in 7.5% (29/385) of patients with PsV or PsA (Table 8). The reasons for treatment interruption included patient request (*n* = 10), symptom improvement (*n* = 7), and AE (*n* = 5). The reasons for resuming treatment included patient request (*n* = 13), symptom worsening (*n* = 3), and recovery from AEs (*n* = 3). The median duration of interruption at any point between the 2nd and 7th injections was 101.0 days. Treatment interruption was reported in 0.0% of patients with GPP or EP.

**TABLE 8:** Treatment interruption in patients with PsV or PsA (safety analysis set).

	PsV (*n* = 310) or PsA (*n* = 75)
Number of episodes of treatment interruption	33
Number of episodes of treatment interruption per patient, median (min–max)	1.0 (1–3)
Patients with at least one treatment interruption	29^a^ (7.5)
Reasons for treatment interruption^b^	
Patient's request	10
Symptom improvement	7
AEs	5
Other	7
Patients with at least one treatment resumption	29 (7.5)
Reasons for treatment resumption^c^	
Patient's request	13
Symptom worsening	3
Recovery from AEs	3
Other	9
Unknown	2
Duration of interruption, days, median (min–max)	
Any point between 2nd and 7th (*n* = 28)	101.0 (66–308)
1st to 2nd (*n* = 5)	91.0 (56–153)
2nd to 3rd (*n* = 4)	119.0 (66–308)
3rd to 4th (*n* = 7)	105.0 (70–196)
4th to 5th (*n* = 6)	119.0 (91–154)
5th to 6th (*n* = 8)	91.5 (77–140)
6th to 7th (*n* = 3)	84.0 (66–84)

*Note*: Values are given as *n* or *n* (%) unless otherwise noted.

Abbreviations: AEs, adverse events; PsA, psoriatic arthritis; PsV, psoriasis vulgaris.

^a^ PsV (*n* = 24) and PsA (*n* = 5).

^b^ Multiple episodes of treatment interruption with the same reason are counted only once per patient.

^c^ Multiple episodes of treatment resumption with the same reason are counted only once per patient.

In patients with PsV or PsA and treatment interruption, the PASI score at each time point during the observation period was significantly lower than the baseline value (mean change from baseline between −5.95 and −9.14; Table S3). Mean PASI scores before and after treatment interruption were also significantly lower than the baseline value (mean change from baseline of −9.25 and −5.58).

## 4 DISCUSSION

In this prospective, post‐marketing surveillance study, guselkumab was well‐tolerated and effective in Japanese patients with psoriasis. During the 52‐week observation period, 8.4% (35/416) of patients experienced ADRs, and 2.9% (12/416) experienced serious ADRs. In the effectiveness analysis set of 236 patients with PsV or PsA, PASI 75, 90, and 100 response rates (95% CI) at week 52 were 69.9% (61.0%–77.9%), 54.5% (45.2%–63.5%), and 32.5% (24.4%–41.6%), respectively. Bio‐naïve patients had a better response to treatment with guselkumab than bio‐experienced patients.

Compared with the previous phase 3 studies in Japan, the mean age of patients in this study was higher for those with PsV or PsA (57.8 or 58.2 vs 47.8 [guselkumab 100 mg group]^9^) and GPP (58.8 vs 42.6^10^), but not for those with EP (55.8 vs 54.6^10^). Disease duration in this study was similar to the previous phase 3 studies for PsV or PsA (mean: 13.81 years or 15.07 years vs 14.39 years [guselkumab 100 mg group]^9^), shorter for GPP (median: 7.25 years vs 14.9 years^10^) and longer for EP (median: 14.00 years vs 5.0 years^10^). Mean baseline PASI scores in this study were lower than those in the previous phase 3 studies for PsV or PsA (12.11 or 9.57 vs 26.73 [guselkumab 100 mg group]^9^), GPP (8.40 vs 29.3^10^), and EP (30.37 vs 40.9^10^). These results are consistent with previous findings that patients in a real‐world setting tend to be older and have slightly longer disease duration and lower baseline PASI scores than those participating in clinical trials, given the strict inclusion criteria and treatment washout requirements.^20^, ^21^ The proportions of male patients with PsV, PsA, or EP in this study were also similar to those in previous phase 3 studies. However, the percentage of male patients in this study was lower among those with GPP (34.6% vs 60.0%^10^). Data from the Japanese Psoriasis Society registry show that GPP is more common in female than male patients (male‐to‐female ratio of 1:1.2), although data from the Ministry of Health, Labour and Welfare show a slightly higher prevalence of GPP in male than female patients (male‐to‐female ratio of 1:0.88).^22^ Given this variation in the male‐to‐female ratio among patients with GPP across studies, more data are needed to better characterize this trend.

No new safety findings were identified in this study, and the safety results were consistent with the well‐characterized safety profile of guselkumab in its approved indications. No patient in this study developed tuberculosis, consistent with previous pooled analyses across multiple clinical trials which also did not identify any cases of active tuberculosis after up to 5 years of treatment with guselkumab.^23^, ^24^ The univariable logistic regression analyses identified a history of a prior medical condition, comorbidity, allergy, and concomitant treatment as predictors of ADRs, but these were not significant in the multivariable logistic regression analysis.

In this study, the PASI and DLQI scores of patients with PsV or PsA improved after the first guselkumab injection and remained lower than baseline values through to week 52. The PASI 75, 90, and 100 response rates at week 52 were 69.9%, 54.5%, and 32.5% in this study. These were lower than the 90.5%, 77.8%, and 47.6% reported for the guselkumab 100 mg group in the previous phase 3 study in Japan,^9^ but comparable to the 78.4%, 62.9%, and 40.4% reported in the real‐world PERSIST study in Germany.^25^ The achievement of a DLQI score of 0/1 at week 52 was 56.9% in this study, which was lower than the 76.7% rate (among patients with a baseline DLQI score >1) in the previous phase 3 study in Japan^9^ but comparable to the 64.6% rate in the PERSIST study.^25^

Our study also demonstrated improved clinical parameters after guselkumab treatment in patients with GPP or PsA in a real‐world setting. Of the 13 patients with GPP in our study, five achieved a CGI rating of “very much improved”, “much improved”, or “minimally improved” at week 52, which confirmed the efficacy of guselkumab demonstrated in 10 patients with GPP in a previous phase 3 study.^10^ Guselkumab treatment led to improvement in the DAS28 (CRP) score in patients with PsA in this study, as in previous phase 3 studies outside Japan.^26^, ^27^ Improvement in a mean PtGA score of more than 10 points, which is considered a clinically meaningful improvement in PsA,^28^, ^29^ was observed, as in a previous phase 3 study in Japan.^9^

Our study showed that patients with higher baseline PASI scores were more likely to achieve PASI 90 responses. A previous registry study of bio‐naïve patients who switched from non‐biologic systemic treatment to biologic treatment also found that having a higher PASI score before switching was associated with a greater overall PASI response.^30^ This association between baseline PASI scores and response to guselkumab may account for the lower PASI 90 response rate at week 52 in our study, given the lower baseline PASI scores compared with the previous phase 3 study.^9^

We also observed that the possibility of achieving a PASI 90 response was lower for patients with comorbidity compared with those without (OR 0.440; 95% CI 0.227–0.852; *p* = 0.015). A previous report from the CorEvitas Psoriasis Registry demonstrated that the likelihood of achieving a PASI 75 response at 6 months of treatment with biologic therapy was 18% lower (OR 0.82; 95% CI 0.67–1.00) among patients with one cardiometabolic comorbidity and 23% lower (OR 0.77; 95% CI 0.63–0.96) among those with two or more other conditions compared with those with none, following adjustment for obesity and other covariates.^31^ Given that 32.2% (76/236) of patients with PsV or PsA in our study had at least one cardiometabolic comorbidity, and 55.9% (132/236) had any comorbidity, these factors may contribute to a poorer response to guselkumab treatment.

In our study, bio‐naïve patients had higher PASI 75 and PASI 90 response rates than bio‐experienced patients over 52 weeks. The possibility of achieving a PASI 90 response tended to be lower in bio‐experienced patients than bio‐naïve patients (univariable analysis, OR 0.526; 95% CI 0.313–0.885; multivariable analysis, OR 0.878; 95% CI 0.455–1.696). The PASI 100 response rates after week 20 were comparable between the bio‐naïve and bio‐experienced groups, which may have been related to patients who discontinued treatment. Up to week 20, treatment was discontinued due to symptom improvement (three bio‐naïve patients and no bio‐experienced patients), insufficient effect (six bio‐naïve patients and 12 bio‐experienced patients), and no‐show (nine bio‐naïve patients and no bio‐experienced patients), suggesting that by week 20, both bio‐naïve patients who responded well to guselkumab and bio‐experienced patients who did not respond well to guselkumab were more likely to discontinue treatment.

In another phase 3b study to evaluate the impact of early intervention with guselkumab in patients with psoriasis (the GUIDE study), those with a disease duration of ≤2 years were more likely to achieve an absolute PASI score of 0 (51.8% vs 39.4%) at week 28 and complete skin clearance at both weeks 20 and 28 (i.e., super‐response; 43.7% vs 28.1%) than those with disease duration of >2 years.^32^ Our study found a positive correlation between disease duration and change in PASI score from baseline in patients with disease duration of <5 years, suggesting the potential benefit of early use of guselkumab for psoriasis. Overall, our findings are consistent with the recent understanding that early intervention may lead to a better disease trajectory in chronic inflammatory conditions.^33^

In a retrospective cohort study of drug survival of IL‐17 and IL‐23 inhibitors, guselkumab demonstrated a higher cumulative probability of drug survival than other biologic agents (0.90 at 24 months and 0.88 at 36 months).^34^ In a systematic review and meta‐analysis, guselkumab yielded a cumulative probability of drug survival of 0.87 after 1 year, 0.81 after 2 years, 0.77 after 3 years, and 0.75 after 5 years.^35^ The treatment discontinuation rate at week 52 in our study was 26.8% (103/385) in patients with PsV or PsA, with rates of 28.6% (50/175) for bio‐experienced patients and 25.2% (53/210) for bio‐naïve patients. This is in line with earlier studies identifying previous exposure to biologic agents as a significant predictor of drug discontinuation.^34^, ^35^ Compared with bio‐experienced patients, bio‐naïve patients were more likely to discontinue treatment because of symptom improvement or not attending a follow up visit and had a lower mean PASI score at treatment discontinuation (3.18 vs 5.68). This suggests that some bio‐naïve patients might discontinue guselkumab despite responding to treatment, which could have a negative impact on overall drug survival.

Finally, 7.5% (29/385) of patients with PsV or PsA experienced at least one episode of treatment interruption during the 52‐week observation period in our study. The number of patients analyzed is relatively small, but PASI scores at week 52 were significantly lower than those at baseline. Among patients in the VOYAGE 2 study who achieved a PASI 90 response at week 28, then withdrew from guselkumab treatment and lost response, 80.4% (41/51) re‐achieved a PASI 90 response after 20 weeks of retreatment with guselkumab.^36^ Of patients who achieved a super‐response with guselkumab treatment in the GUIDE study, 92.6% (137/148) and 91.9% (137/149) had an absolute PASI response of <3 at week 68 with every 8 week (Q8W) and every 16 week (Q16W) dosing, demonstrating non‐inferiority of Q16W vs Q8W for maintaining disease control among super‐responders.^37^ These findings support the fact that even if treatment is interrupted, clinical response may be maintained or re‐achieved after resuming treatment with guselkumab. Considering that some patients may need to withdraw or interrupt chronic treatment with biologic agents, further studies are necessary to better understand patient characteristics that may allow for safe pauses in treatment or dosing interval extension without substantially compromising clinical effect.

This study had some considerable strengths, particularly that it was a prospective, multicenter, large‐scale, real‐world survey that provided valuable insights on the effectiveness and safety of guselkumab in Japanese patients with psoriasis, including GPP, with various comorbidity and previous treatment experiences. However, its limitations must be acknowledged. First, this was a non‐interventional, observational, post‐marketing, surveillance study without a control group, and may have been subject to selection bias and loss‐to‐follow‐up bias. Second, DAS28 (CRP) and PtGA scores were assessed in less than one‐third of the patients with PsA in this study. The effectiveness of guselkumab in treating PsA needs to be assessed further in larger numbers of patients in other settings. Third, each investigator determined whether treatment was interrupted, and the impact of treatment interruption on guselkumab effectiveness was assessed in a limited population. Fourth, we conducted an “as observed” analysis without imputing missing data. Although missing data occurred randomly in this study, generalization from our findings should proceed cautiously.

In conclusion, this prospective, post‐marketing, surveillance study demonstrated that guselkumab was well‐tolerated and effective in Japanese patients with psoriasis in a real‐world setting. No new safety concerns were identified. Bio‐naïve patients and patients without comorbidity achieved particularly high levels of response with guselkumab treatment in this study, which supports the early use of guselkumab in those with recent onset psoriasis.

## CONFLICT OF INTEREST STATEMENT

Yayoi Tada has received research grants, collaborative research funding, and Speaker Fees from Maruho Co., Ltd., Eisai Co., Ltd., Nippon Boehringer Ingelheim Co., Ltd., Janssen Pharma K.K., UCB Japan Co., Ltd., Mitsubishi Tanabe Pharma Corporation, Torii Pharmaceutical Co., Ltd., AbbVie GK, Kyowa Kirin Co., Ltd., Sun Pharma Japan Limited, Amgen K.K., Eli Lilly Japan K.K., Taiho Pharmaceutical Co., Ltd., and Bristol‐Myers Squibb K.K., and has served as a consultant for Janssen Pharmaceutical K.K. Keiichi Yamanaka has received speaker fees from Eli Lilly Japan K.K., Janssen Pharmaceutical K.K., AbbVie GK, Sanofi K.K., Eisai Co., Ltd., Nippon Boehringer Ingelheim Co., Ltd., Bristol‐Myers Squibb K.K., Teikoku Seiyaku Co., Ltd., Mitsubishi Tanabe Pharma Corporation, Maruho Co., Ltd., Novartis Pharma K.K., Torii Pharmaceutical Co., Ltd., Pfizer Japan Inc., Leo Pharma, Taiho Pharmaceutical Co., Ltd., Nippon Kayaku Co., Ltd., Sun Pharma Japan Limited, UCB Japan Co., Ltd., Celgene Corporation, Kaken Pharmaceutical Co., Ltd. and has received Grants‐in‐Aid for Scientific Research Japan, Maruho Co., Ltd., Torii Pharmaceutical Co., Ltd., Taiho Pharmaceutical Co., Ltd., Sasaki Chemical Co., Ltd., Kaken Pharmaceutical Co., Ltd., Nippon Kayaku Co., Ltd., Otsuka Pharmaceutical Co., Ltd., Nihon Pharmaceutical Co., Ltd., Sun Pharma Japan Limited, and has served as a consultant for Janssen Pharmaceutical K.K. The other authors are full‐time employees of Janssen Pharmaceutical K.K., Tokyo, Japan. Keiichi Yamanaka is an Editorial Board member of *The Journal of Dermatology* and an author of this article. To minimize bias, he was excluded from all editorial decision‐making related to the acceptance of this article for publication.

## ETHICS STATEMENT

The protocol and ethical considerations of the study were approved by the internal review board of each participating institution.

## INFORMED CONSENT STATEMENT

The study was conducted in accordance with Good Post‐marketing Study Practice (GPSP, the Ministry of Health, Labour and Welfare Ordinance No. 171., dated December 20, 2004), which does not require patient consent for post‐marketing surveillance studies.

Registry and the registration no. of the study/trial: University Hospital Medical Information Network Clinical Trials Registry UMIN000032969.

## Supporting information


Tables S1‐S3.
