# Supplementary material for: Real‐world safety and effectiveness of guselkumab in patients with psoriasis: A post‐marketing surveillance study through up to week 52 in Japan
Source: J Dermatol. 2025 Mar 29;52(6):967–82. doi: 10.1111/1346-8138.17710 (PMC12149365; doi:10.1111/1346-8138.17710)
Supplement: Supplementary file 1 — Tables S1‐S3. [file JDE-52-967-s001.docx]

**SUPPORTING INFORMATION**

**Supplementary Tables**

**Table S1.** Prevalence of comorbidities in patients with PsV or PsA, stratified by years of disease duration (effectiveness analysis set)

|  | n | Any comorbidity | Cardiometabolic comorbidities^‡^ |
| --- | --- | --- | --- |
| Patients with PsV or PsA | 236 | 132 (55.9) | 76 (32.2) |
| Disease duration, years |  |  |  |
| < 10 | 79 | 33 (41.8) | 19 (24.1) |
| ≥ 10 and < 20 | 81 | 47 (58.0) | 22 (27.2) |
| ≥ 20 and < 30 | 49 | 31 (63.3) | 20 (40.8) |
| ≥ 30 | 23 | 17 (73.9) | 12 (52.2) |
| Unknown | 4 | 4 (100.0) | 3 (75.0) |
| *p* value^†^ |  | 0.002 | 0.004 |

Values are given as n (%).

^†^Cochran–Armitage trend test (“Unknown” were excluded).

^‡^Hypertension, diabetes mellitus, hyperuricemia, dyslipidemia, or hyperlipidemia.

PsA, psoriatic arthritis; PsV, psoriasis vulgaris

**Table S2-1**. DAS28 (CRP) scores in patients with PsA (effective analysis set)

|  | DAS28 (CRP) score | | | | |
| --- | --- | --- | --- | --- | --- |
|  | Observed value | | Change from baseline | | |
|  | n | mean (SD) | n | mean (SD) | *p* value^†^ |
| Week 0 | 15 | 3.10 (1.02) | - | - | - |
| Week 20 | 12 | 2.79 (1.31) | 11 | −0.69 (1.01) | 0.046 |
| Week 52 | 6 | 2.34 (1.00) | 4 | −0.71 (1.93) | 0.517 |
| LOV | 16 | 2.62 (1.10) | 13 | −0.63 (1.08) | 0.058 |

^†^Paired *t*-test.

CRP, c-reactive protein; DAS28, disease activity score 28; LOV, last observed value; PsA, psoriatic arthritis; SD, standard deviation

**Table S2-2**. PtGA scores in patients with PsA (effective analysis set)

|  | PtGA score | | | | |
| --- | --- | --- | --- | --- | --- |
|  | Observed value | | Change from baseline | | |
|  | n | mean (SD) | n | mean (SD) | *p* value^†^ |
| Week 0 | 18 | 45.22 (30.25) | - | - | - |
| Week 20 | 15 | 34.93 (27.13) | 13 | −25.00 (29.81) | 0.011 |
| Week 52 | 9 | 23.56 (27.99) | 7 | −22.57 (41.44) | 0.200 |
| LOV | 19 | 29.32 (25.78) | 16 | −18.81 (28.18) | 0.017 |

^†^Paired *t*-test.

LOV, last observed value; PsA, psoriatic arthritis; PtGA, patient global assessment of disease activity; SD, standard deviation

**Table S3.** PASI scores in patients with PsV or PsA and treatment interruption (effectiveness analysis set)

|  | PASI score | | | | |
| --- | --- | --- | --- | --- | --- |
|  | Observed value | | Change from baseline | | |
|  | n | mean (SD) | n | mean (SD) | *p* value^†^ |
| Time point |  |  |  |  |  |
| Week 0 | 18 | 10.76 (9.97) | - | - | - |
| Week 4 | 15 | 5.30 (5.40) | 15 | −6.07 (8.83) | 0.019 |
| Week 12 | 15 | 3.23 (3.26) | 15 | −7.53 (10.26) | 0.013 |
| Week 20 | 11 | 4.81 (5.65) | 11 | −5.95 (6.37) | 0.011 |
| Week 36 | 8 | 2.28 (3.03) | 8 | −8.03 (5.45) | 0.004 |
| Week 52 | 6 | 1.78 (1.61) | 6 | −8.97 (5.76) | 0.012 |
| LOV | 18 | 1.62 (2.10) | 18 | −9.14 (9.48) | < 0.001 |
| Before and after interruption | |  |  |  |  |
| Before interruption | 16 | 4.21 (5.94) | 13 | −9.25 (9.44) | 0.004 |
| After interruption | 12 | 3.83 (4.88) | 12 | −5.58 (5.66) | 0.006 |

^†^Paired *t*-test.

LOV, last observed value; PASI, psoriasis area and severity index; PsA, psoriatic arthritis; PsV, psoriasis vulgaris; SD, standard deviation
